# How Australia's social media minimum age law may reshape research recruitment for adolescents under 16

**DOI:** 10.1016/j.lanwpc.2026.101822

**Published:** 2026-03-05

**Authors:** Bridianne O’Dea, Isobelle McKenzie, Michelle Torok

**Affiliations:** a Flinders University Institute for Mental Health and Wellbeing, College of Education, Psychology and Social Work, Flinders University, Bedford Park, South Australia, Australia; b Black Dog Institute, Faculty of Medicine and Health, University of New South Wales, Sydney, New South Wales, Australia

**Keywords:** Adolescent, Research subject recruitment, Social media, Informed consent, Health policy

## Abstract

Social media is a popular recruitment tool for adolescent research as it can enable rapid, low-cost, and targeted enrolment. Australia's Online Safety Amendment Bill, which introduced a mandatory minimum age of 16 from December 2025, is likely to disrupt some researchers' ability to recruit adolescents aged 13–15 who are commonly accessed through these platforms. This viewpoint examines the implications of the legislation for recruitment feasibility, sample representativeness, and research equity in Australia, with flow-on effects for international studies. We identify three areas of impact: shifts in sample composition due to reduced reach; increased reliance on gatekeeper-mediated recruitment, with potential risks to participation privacy, and sample diversity; and greater operational complexity, longer timelines, and higher costs. We highlight opportunities to strengthen youth-centred research infrastructure, including establishing a national youth research registry, supporting multi-modal recruitment through expanded funding and timelines, and advancing ethical, developmentally appropriate consent pathways that minimise gatekeeping.

Many researchers rely on social media to recruit adolescents for research because it can enable highly targeted, rapid and low-cost enrolment across diverse study designs.^1^, ^2^, ^3^ Platform affordances, such as targeted algorithms and user-sharing functions, have increased the reach of research recruitment to young people, including to diverse and hard-to-engage youth.^4^ Young people also report preferring social media as a recruitment pathway.^5^ However, Australia's Online Safety Amendment (Social Media Minimum Age) Bill introduces a mandatory minimum account age of 16 on designated social media platforms from 10 December 2025.^6^ The age restriction applies to platforms whose sole or primary purpose is to enable online social interaction between two or more users. Platforms currently deemed as age-restricted under this new legislation in Australia include Facebook, Instagram, Kick, Reddit, Snapchat, Threads, TikTok, Twitch, X, YouTube, BlueSky, Match Services (e.g. Tinder, Hinge), Yubo, Wizz, Lemon8, and Bigo Live, but may expand with future assessments.^7^ This has direct implications for researchers who rely on age-restricted social media platforms to recruit early-to-mid stage adolescents (13–15 years), with likely effects on participation rates, recruitment duration, costs, representativeness, and scale. Recruitment strategies will therefore need significant adaptation. This viewpoint outlines some of the anticipated effects of the age-restriction policy on research recruitment in Australia and on the representation of younger Australian adolescents in international studies. We offer some pragmatic, forward-looking recommendations to help researchers prepare for this change.

## Anticipated effects of social media restrictions on research recruitment

### Shifts in sample composition and selection bias

Social media is often a central recruitment channel for many types of adolescent research because it can enable rapid, low-cost access to diverse youth.^1^^,^^8^, ^9^, ^10^, ^11^ Reviews have found that the majority of human research studies and clinical trials use social media for recruitment, with 45–58% relying on it as the sole strategy.^12^, ^13^, ^14^ The Social Media Minimum Age Bill will materially restrict or eliminate these channels for youth recruitment, shrinking the accessible pool and altering who can be reached. Although social media recruitment has resulted in some sample biases and mistargeted recruitment due to misrepresented profile information,^10^^,^^15^ the introduction of age-restrictions is likely to further skew youth recruitment towards specific subgroups.

Changes in social media access may mean that adolescent samples now increasingly include (i) older youth (≥16 years), (ii) youth who use social media platforms that are not currently age-restricted under the legislation but do have advertising functionality (e.g. gaming and coding platforms such as Discord, Steam, GitHub) and (iii) youth reachable through parents/guardians, schools, health services, or community organisations, which may compromise the external validity of some research and limit the involvement of diverse groups (e.g. those defined by cultural and gender identities, sexuality, disability, health status, and geographic location).^16^ These factors may then exacerbate disparities in whose data informs clinical guidelines and policy, emphasising the importance of researchers proactively planning for, monitoring, and measuring changes in sample composition.

To account for the impact of the age-restrictions, researchers will need to transparently document any changes in recruitment pathways, pre-specify planned adaptations and seek ethics approval prior to use, as well as report differences in enrolment rates, demographic profiles, and attrition by channel once implemented. While recruiting through non-age-restricted platforms may help maintain adolescent participation rates, it may introduce distinct methodological and ethical challenges, requiring researchers and ethics committees to develop, evaluate, and approve new recruitment processes. Pre-post analyses of sample compositions recruited before and after the legislation may clarify how the policy affects recruitment feasibility and representativeness. Bias-mitigation strategies, such as stratifying recruitment targets across modes, enhancing community partnerships, and developing supplementary offline recruitment pathways, will become increasingly important to achieve sample diversity and protect the validity and integrity of research involving Australian adolescents.

### Increased reliance on alternative consent pathways and adult gatekeepers

For under-16 participants, researchers may pivot to parent/guardian mediated recruitment. Although effective in many studies, active parental consent often reduces adolescent participation and disproportionately excludes lower socio-economic groups.^17^ Parent-focused recruitment may be slower, requiring time to build awareness, trust, and buy-in. It also relies on parents/guardians' research literacy and engagement, which may bias samples toward higher-resource families and limit adolescents' privacy when self-initiating participation. This is particularly problematic for sensitive topics, such as mental health, gender identity and sexual health, where recruitment is already challenging^14^ and adolescents are reluctant to disclose their interest to parents/guardians or caregivers. Researchers targeting parents/guardians, teachers or clinicians, must account for relational power dynamics to avoid inadvertent coercion when recruitment passes through authority figures.^16^^,^^17^ To ensure ethical and effective recruitment, researchers need to understand how gatekeepers communicate study information to adolescents, yet may lack the time, scope, or resources to observe these interactions. This limits researchers’ ability to refine recruitment materials and prevents insights into why recruitment efforts may have been unsuccessful.

### Operational complexity and cost increases

Social media platforms can offer cost-effective recruitment pathways, with almost double the odds of successful recruitment per dollar spent compared with non-social media methods.^18^ Many studies operate on constrained budgets and compressed timelines and rely on rapid recruitment to achieve sample sizes and have used social media advertisements as a first-line recruitment method due to its operational advantages. Thus, without social media, recruitment periods may lengthen, samples may be under-powered, and per-participant costs may increase. Alternative recruitment approaches that use community outreach, school partnerships, and health services can require greater personnel time, multiple levels of ethical and institutional approval across several sites and organisations, expanding costs and administrative workloads. These factors challenge the feasibility of national youth recruitment in regions like Australia, where multi-site and multi-state education and health ethics approval processes are required. Social media also allowed some research recruitment to continue during the COVID-19 pandemic when in-person contact was disrupted by service and school closures.^19^ Losing this pathway reduces resilience of some research operations. However, protecting research integrity when using social media for recruitment has become a challenge for researchers, particularly in incentivised studies that rely on participant honesty to determine eligibility.^20^, ^21^, ^22^ Managing fraudulent participation has increased workloads and costs and threatened the quality and integrity of research findings.^21^^,^^22^ Taken together, these issues underscore the need for a critical re-evaluation of social media recruitment for youth.

## An opportunity

The introduction of the Social Media Minimum Age Bill in Australia has created an inflection point for youth-centric research, and there is now an opportunity to drive forward a practice change that has global relevance, given the high likelihood that similar policies will be introduced internationally. We argue that some of the barriers to research recruitment arising from age-restricted platforms can be mitigated by considering the following.

### Build a national, centralised youth research registry co-designed with young people

Developing a dedicated, national participant registry that includes adolescents under 16 could increase awareness of, and access to, research opportunities in the absence of social media. Existing models demonstrate feasibility: for example, Neuroscience Research Australia's (NeuRA) Child and Adolescent Healthy Volunteer Registry is used for recruiting children and adolescents up to 16 years with parental involvement, annual re-consent, and provides a limit of three research invitations per year to keep participation voluntary and manageable. Similarly, the Australian Child Neurodevelopment Registry promotes participation in studies involving children with developmental conditions, while The Australian National Centre for Action on Child Sexual Abuse maintains a research register for studies involving children and adolescents. Many adolescent research groups, including our own, have also established small-scale internal registries that contain the details of adolescents who have agreed to be contacted for research. However, separate registries risk duplication and excessive contact. Building awareness and engagement of separate registries is also slow and requires considerable investment from organisations that operate within resource-constrained, not-for-profit environments. We argue that a government funded, nationally coordinated, multi-institutional youth research registry co-designed with young people that respects their autonomy is critical for ensuring equitable access to research participation for Australian adolescents. A model like the ‘Join Us’ research register for adults, developed by The George Institute at the University of New South Wales, similar to the ‘Be Part of Research’ initiative in the United Kingdom, could be expanded to include individuals under 16, connecting young people and families with research projects aligned to their interests while ensuring robust ethical safeguards and allowances for parental involvement and consent. The registry could also support age and identity verification processes to reduce fraudulent or imposter participation, improving the integrity of youth research more broadly.

A national registry would require infrastructure funding and operational support from governments to enable ethics-approved personal data collection, high-level data security, and dedicated staffing. A blended, not-for-profit funding model that includes government support and streamlined processes for researchers to use grant funds to promote studies on the registry could enhance sustainability, reduce administrative burden, and ensure consistent quality assurance while also supporting no-cost and equal access for unfunded studies. Importantly, promotion of the registry should embed principles of cultural safety, equitable inclusion, and accessibility, ensuring registrations and research opportunities are not limited to high-resource individuals or communities and include young people from diverse backgrounds and gender identities. Transparent monitoring and reporting of the registry's demographic profile will be essential for identifying and addressing sample bias. To date, the ‘Join Us’ register has enrolled more females (∼70%) than males, with greater representation of younger (18–35 years; 27%) and older (>50 years; 51%) adults compared with those of middle age.^23^ In contrast, participation rates for culturally and linguistically diversity individuals (20%) and First Nations peoples (3%) are broadly consistent with Australian population metrics.^23^, ^24^, ^25^ Establishing clear governance structures including youth advisory groups, First Nations leadership, and transparent data stewardship arrangements would further strengthen trust and participation. Such a registry represents a major national research infrastructure investment that can be leveraged across institutions, disciplines, and research groups, supporting more efficient recruitment, reducing duplication, and improving representation in youth research.

### Greater allowances in funding and project timelines to support multi-modal recruitment strategies

Although multi-pronged recruitment across youth settings can enhance adolescent participation,^14^ building and maintaining these channels requires significant time, relationship-building, and administrative capacity. Research teams typically operate within frugal budgets and compressed timelines, and many community organisations lack the staff time, financial resources or institutional support to take on recruitment roles without dedicated funding. This places considerable pressure on finite grant budgets and can result in limited reach, inconsistent recruitment efforts, and variable quality of information provided to participants.^26^ Delegating participant contact to busy frontline staff, such as school teachers, administrative personnel, health professionals, or youth workers, can undermine recruitment efforts as competing priorities, reduced motivation, and poor understanding of study aims may limit engagement with recruitment activities.^26^ These factors often prolong recruitment timelines, decrease yield, and introduce variability in how studies are described to young people. Stakeholder mapping can help researchers identify appropriate recruitment partners by clarifying where young people spend time and which organisations they trust, noting that setting-based recruitment (e.g. schools, clinics) is only effective when the target sample naturally engages with those settings.

Recruitment and retention frameworks, such as the Tailored Panel Management,^27^ can guide structured, multi-level recruitment planning. However, implementing iterative and flexible adaptations is challenging when multiple partners are involved and ethics review cycles delay timely updates to materials and procedures. Hybrid strategies, such as using electronic health records to identify eligible participants followed by text message or postal invitations to parents/guardians, have shown promise for enrolling younger adolescents under 16 years and other hard-to-reach groups.^28^^,^^29^ School-based recruitment can also yield large, general population samples and capitalise on peer networks to support snowball sampling.^3^^,^^5^^,^^30^^,^^31^ Our own programs have successfully recruited more than 8000 adolescents into research studies through coordinated school partnerships.^32^^,^^33^ However, school-based approaches are ineffective for youth disengaged from education, and can be challenging for low-prevalence health conditions, and other subgroups.^29^^,^^34^ Given these limitations, multimodal recruitment strategies are essential to build more durable and resilient sampling frames that can withstand policy shifts like restrictions to social media without compromising sample diversity or representativeness. Sustained investment from funders is therefore critical, including greater funding allowances for partnership development, staff time for community engagement, flexible timelines, and top-up funding structures that support ongoing adaptation throughout the recruitment period.

### Advance ethical and privacy-preserving consent models that minimise gatekeeping

The Social Media Minimum Age Bill also presents an opportunity to reconsider, update and strengthen consent models for research involving minors, ensuring legal requirements are balanced with adolescent autonomy and appropriate parent/guardian involvement. Consent practices vary widely across countries and research areas, with differences in the minimum age for parental consent and the modes of consent permitted.^17^ We have successfully used Gillick Competence measures in several studies with adolescents under 18 to assess their understanding of the research.^33^^,^^35^ These non-standardised measures involve interactive activities (e.g. online questionnaires) designed to assess a young person's capacity to consent by examining their comprehension and understanding of the study information sheet and consent form. Validating this approach across diverse study designs, age groups, and cultural contexts would help clarify when Gillick-based consent is appropriate, when it is insufficient and where additional safeguards are required. Expanding the evidence base for the validity and effectiveness of competence-based consent, while examining its developmental, ethical, and contextual boundaries, may create more flexible participation pathways for adolescents under 16 without compromising ethical standards. In parallel, a deeper understanding of adolescents' experiences of research participation can support ethical guidelines that better enable equitable youth involvement. Strengthening the capacity of human research ethics committees is also essential. Providing training on adolescent cognitive and developmental capacities, and on the value of youth partnership in study design, may improve committee decision-making and support proportionate risk-benefit assessments for adolescent research.^17^ Given that communication with parents/guardians is associated with adolescents' willingness to enrol in research,^36^ rigid default reliance on parental consent may inadvertently limit participation. This reinforces the need for tailored, context-sensitive approaches to recruitment and consent that respect adolescent autonomy while ensuring appropriate parental engagement.

## Concluding remarks

The Social Media Minimum Age legislation will disrupt a widely used recruitment mode for adolescent research in Australia. The consequence is not simply operational inconvenience: it may threaten representativeness, increase recruitment costs and time, and require ethically sensitive redesign of consent and outreach pathways. As several other nations consider introducing similar legislation, the implications for adolescent research are likely to be far-reaching. However, the policy also invites a reset: an opportunity to build more resilient, diversified recruitment strategies. To adapt, the research community should: (1) support the establishment of national youth research registry in Australia and other regions as required; (2) advocate for increased funding and extended project timelines to account for slower, labour-intensive recruitment (3) advance ethical, developmentally appropriate consent pathways that minimise gatekeeping and prioritise equity. Researchers, funders, and ethics bodies must work collaboratively to minimise unintended impacts of the legislation on research methods and to ensure that research participation remains accessible and inclusive of younger adolescents while respecting the public health rationale that motivated the policy. In practice, this could be supported through a targeted, collaborative funding call to establish consortia comprising adolescents and their parents/guardians, researchers, institutional ethics committees, and funders to systematically examine how social media restrictions affect research participation among adolescents under 16. Evaluating downstream effects of this legislation on research participation will be essential to inform mitigation strategies and enable the global research community to response proactively to similar policy developments.

## Contributors

BOD, MT, and IM conceptualised the work. BOD and IM conducted the literature search and review. BOD wrote the original draft with MT and IM reviewing and editing.

## Declaration of interests

We declare no competing interests.
